# New Spherical Gamma-Ray and Neutron Emitting Sources for Testing of Radiation Detection Instruments

**DOI:** 10.6028/jres.114.022

**Published:** 2009-12-01

**Authors:** L. Lucas, L. Pibida

**Affiliations:** National Institute of Standards and Technology, Gaithersburg, MD 20899-8462

**Keywords:** aluminum sphere, density gauges, gamma-ray emitting sources, irradiators, neutron emitting sources, radiation detection, shielding, uncertainty range

## Abstract

The National Institute of Standards and Technology (NIST) has developed new gamma-ray and neutron emitting sources for testing radiation detection systems. These radioactive sources were developed for testing of detection systems in maritime applications. This required special source characteristics.

## 1. Introduction

NIST designed and built a new type of radioactive source for testing of detection systems in maritime applications. These new sources, designed for testing of radiation detection instruments, need to have specific characteristics that differ from the previous NIST source design for testing of radiation detection systems against different ANSI/IEEE standards for homeland security applications [1, 2, 3, 4]. These new radioactive sources need to meet more demanding requirements due to the environment in which detectors must be tested. For example, as one of the uses includes testing in maritime environments the sources need to float, in case that the sources accidentally fall overboard, and withstand salt water environments without corrosion. Other requirements for these sources include knowing the spectral shape and the angular uniformity of the source emission. Four new gamma-ray emitting sources were built to include ^57^Co, ^60^Co, ^137^Cs and ^133^Ba. The ^57^Co and ^133^Ba sources were designed such that their spectral shape has minimal distortion relative to the emission of a point source. For the ^137^Cs and ^60^Co sources, the spectral shape of point sources was modified to simulate that of ^137^Cs density gauges and ^137^Cs and ^60^Co gamma-ray beam irradiators inside their closed shielding. In addition, a neutron-emitting ^252^Cf source as specified in several ANSI/IEEE standards [2, 3, 4] for testing of radiation detection instruments used in homeland security applications was mounted inside a 15 cm (6 inch) diameter aluminum sphere so that the source will float in water.

## 2. Source Design Considerations

**Fig. 1:**
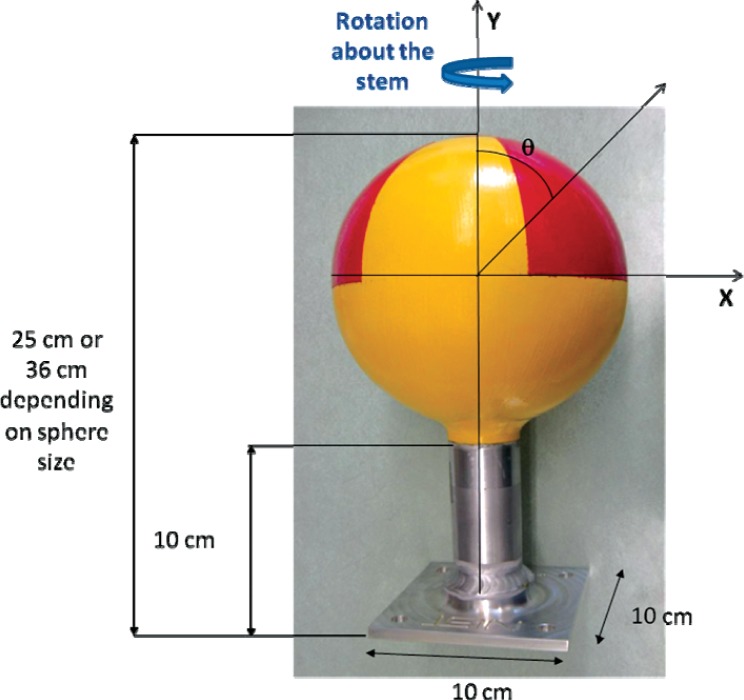
External source dimensions and angles of rotation for measurements.

These radioactive test sources are designed to meet the following requirements:

1. The photon emission rate of the main gamma ray line must be within 20 % of a specified value during the time of use of the source.
2. The photon emission rate of the main gamma ray line must be as spherically uniform as practical. The design goal is to have the photon emission rate of the main gamma ray line be within 10 % (preferably within 5 %) of the average value at the polar angle θ = 90° for all angles from θ = 0 to θ = 135°. The angle θ is measured relative to the top of the sphere, opposite end from the mounting stem, of the designed radioactive source. Therefore, θ = 90° corresponds to the direction perpendicular to the stem while θ = 180° corresponds to the direction parallel to the stem (See Fig. 1).
3. The low-energy part of the photon spectrum must have a shape similar to a specified type of radioactive source (e.g., density gauges, irradiators.)
4. The sources must float in water, must be highly visible, and must be easily retrievable from the water.
5. The sources must be rugged, shock resistant, and not adversely affected by salt water or other substances that they may contact (e.g., gasoline, oils, etc.). The source geometry must not be affected by its orientation, source components should not move inside when mounted in different orientations.
6. The sources must be securely sealed, yet be accessible so that the radioactive material can be removed and reloaded.

## 3. Source Design Measurements

**Fig. 2:**
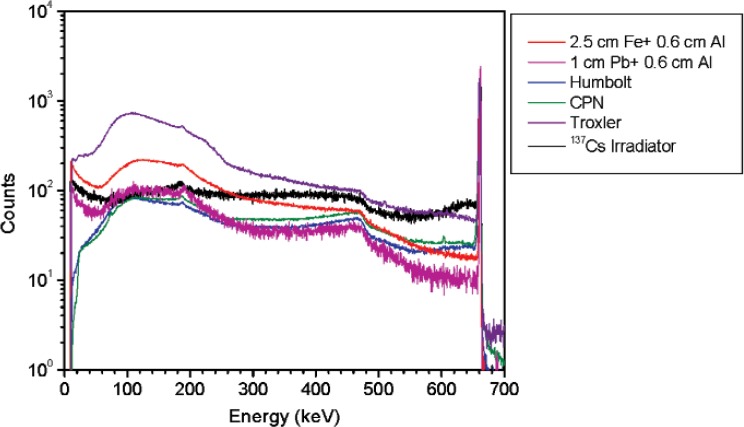
Spectra for the density gauges and the ^137^Cs irradiator together with the ^137^Cs NIST ampoule shielded by iron or lead in addition to 6 mm of aluminum. Spectra are normalized to 10 000 counts in the 662 keV peak areas.

In order to simulate the gamma-ray emission from a density gauge or an irradiator, a 37 MBq (1 mCi) ^137^Cs vial (1 mL glass vial) was shielded by different types and amounts of materials. Spectra from three different density gauge models and several NIST irradiators were acquired. Density gauge measurements were performed using a high purity germanium (HPGe) detector (diameter = 47 mm, length = 38 mm, absorbing layer = 1.27 mm aluminum). The measurements of the density gauges were performed at 65 cm from the source container. The irradiator and ^137^Cs vial measurements were performed using a HPGe L-detector (diameter = 55.2 mm, length = 57.9 mm, absorbing layer = 1.27 mm aluminum, end cap to detector distance = 3 mm). Figure 2 shows a subset of the spectra acquired for the density gauges and one of the irradiators together with the ^137^Cs vial shielded by steel or lead in addition to 6 mm of aluminum. The spectra are normalized to the area of the 662 keV peak. Table 1 lists the density gauges measured. The ^137^Cs irradiator measurements were performed at 5.5 m from the source. The irradiator activity was approximately 3.5 GBq (94 Ci) shielded by approximately 15 cm (6 inches) of lead. All measurements were performed at a distance such that the dead time was less than 2 %; this minimizes random summing.

**Fig. 3:**
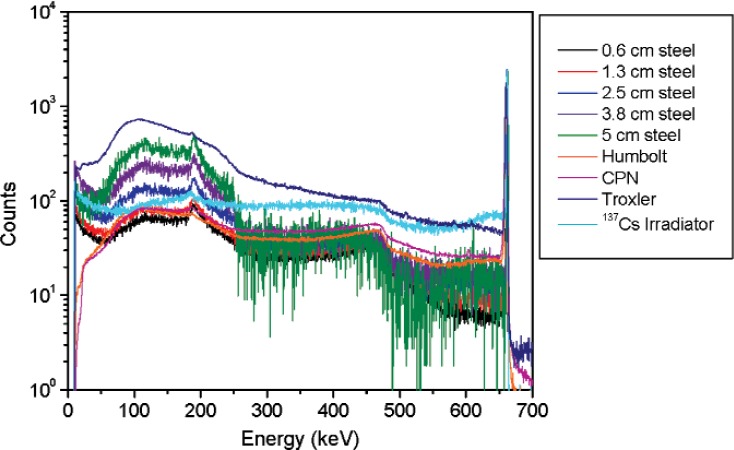
Spectra for the density gauges and the ^137^Cs irradiator together with the ^137^Cs vial shielded by different thicknesses of steel. Spectra are normalized to 10 000 counts in the 662 keV peak areas.

**Fig. 4:**
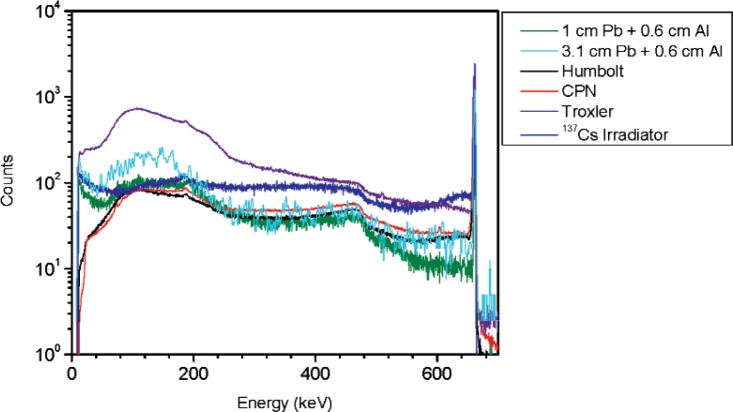
Spectra for the density gauges and the ^137^Cs irradiator together with the ^137^Cs vial shielded by two different thickness of lead. A fixed thickness of 6 mm of aluminum was added to the lead shields. Spectra are normalized to 10 000 counts in the 662 keV peak areas.

Spectra for the ^137^Cs vial were acquired for different shielding materials and thicknesses; the source-to-detector distance was 2.2 m. Several measurements were performed for different steel thicknesses to study the effect on the spectral shape for ^137^Cs. Results are shown in Fig. 3. Similar measurements were performed for different thicknesses of lead (3.1 cm, 2.5 cm, 1.7 cm and 1.0 cm) plus 6 mm of aluminum. Results for the 3.1 cm and 1 cm lead thickness measurements are shown in Fig. 4.

From these measurements it was concluded that the best way of matching the spectral shape of a ^137^Cs irradiator and a density gauge is by adding a thickness of approximately 1 cm of lead and 6 mm of aluminum to a ^137^Cs point source.

**Fig. 5:**
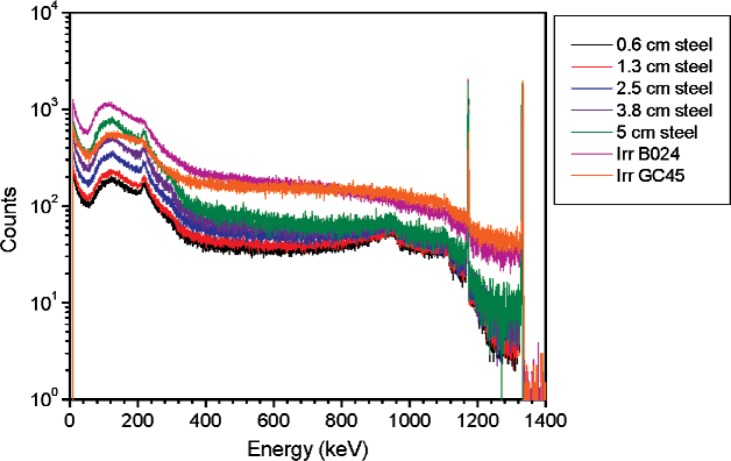
Spectra for the ^60^Co irradiators together with the ^60^Co vial shielded by different thicknesses of steel. Spectra are normalized to 10 000 counts in the 1332 keV peak areas.

**Fig. 6:**
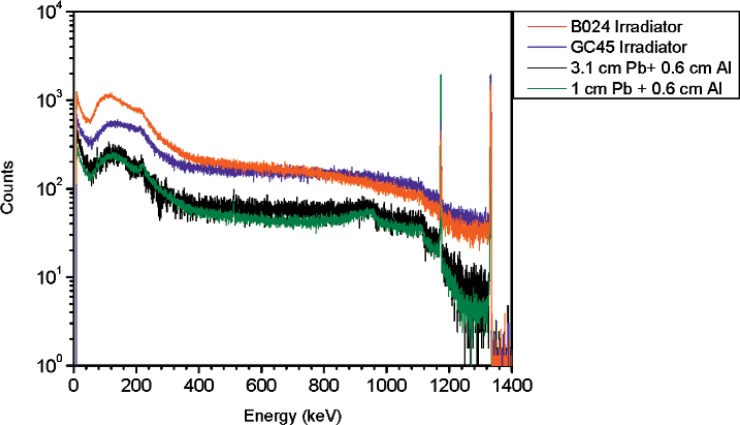
Spectra for the ^60^Co irradiators together with the ^60^Co vial shielded by different thicknesses of lead plus 6 mm of aluminum. Spectra are normalized to 10 000 counts in the 1332 keV peak areas.

For the ^60^Co source, similar measurements were performed to simulate the spectral shape produced by irradiators. There are several ^60^Co irradiators at NIST but due to irradiator location (some of the irradiators are too close to one another) there were only two for which a clean spectrum could be obtained. One of the irradiators is a high intensity therapy level beam irradiator (referred to as B024) and the other is food irradiator (referred to as GC45). The therapy level beam irradiator (B024) is a 500 TBq (13.5 kCi) ^60^Co source (reference date: November 24, 1999) shielded by approximately 23 cm (9 inches) of lead. The HPGe measurements were performed at 1.66 m from the irradiator. The GC45 irradiator is a 29 TBq (777Ci) ^60^Co source (reference date: August 1, 2001) shielded by 25 cm (10 inches) of lead. The HPGe measurements were performed at 62 cm from the irradiator. In order to simulate the gamma-ray emission in the low energy region from an irradiator, a 37 MBq (1 mCi) ^60^Co vial was shielded by different types of materials as well as different amounts of shielding. These measurements were performed at 2.2 m from the source (see Figs. 5 and 6).

From these measurements it was concluded that the spectra of ^60^Co irradiators are best reproduced or simulated by adding a shield of approximately 5 cm (2 inches) of steel and 6 mm of aluminum to a ^60^Co point source.

The ^57^Co and ^133^Ba sources were designed to produce a gamma emission rate equivalent to a bare source activity of 1.67 MBq (45 µCi) and 11.1 MBq (300 µCi) respectively. Therefore, ^57^Co and ^133^Ba point sources were mounted inside thin aluminum holders. The ^252^Cf source was designed to produce a neutron emission rate of 2 × 10^4^ s^−1^ as specified in Ref. [2].

## 4. Source Construction

The ^57^Co and ^133^Ba point sources are made by drying a solution onto glass fiber filter paper 14 mm in diameter and 1 mm thick. This filter paper is then mounted in a 0.3 cm thick aluminum holder shaped to have a uniform gamma-ray emission between 0° and ± 135°. The holder was then mounted inside a 0.15 cm thick aluminum stem welded inside a 0.3 cm thick 15 cm (6 inch) diameter aluminum sphere that is welded shut. The sphere has a 10 cm (4 inch) long aluminum stem attached to hold the source during testing.

In the ^137^Cs source, the radioactive material is contained in a welded cylinder made out of platinum-iridium. The cylinder has a ^137^Cs activity of 185 MBq. The cylinder is mounted inside a 1 cm thick lead absorber that is shaped in such a way as to produce a spherically uniform photon emission rate for the 662 keV gamma-rays. The lead absorber is then mounted inside a 0.15 cm thick aluminum stem welded inside a similar 15 cm diameter aluminum sphere that is welded shut.

The ^60^Co source was built using cobalt pellets that were irradiated in the NIST reactor. The pellets are 2 mm in diameter and 2 mm long. Each pellet contains approximately 28 MBq of ^60^Co. This was achieved by irradiating the cobalt pellets (99.99 + % purity) for 4 hours in a thermal neutron flux of ≈ 3 × 10^13^ s^−l^ cm^−2^. The pellets are then mounted inside a stainless steel rod with a 0.38 cm wall thickness. This rod is then mounted inside a stainless steel sphere that is 4.8 cm in radius. This stainless steel sphere is then mounted inside a 0.3 cm thick 10 cm (4 inch) diameter aluminum sphere that is in turn mounted inside a 0.3 cm thick 25 cm (10 inch) diameter aluminum sphere that is welded shut.

The ^252^Cf source is built using a very small Cf_2_O_3_-palladium wire. The wire is 2 % californium oxide, 98 % palladium and is encapsulated in a stainless steel cylinder with an outside diameter ≈ 5.5 mm, an inside diameter of ≈ 4 mm, an outside length of ≈ 12 mm and an inside length of ≈ 6 mm. This cylinder is mounted inside another steel cylinder such that the total wall thickness is 1 cm. This cylinder is then mounted inside a similar 15 cm diameter aluminum sphere that is welded shut.

**Fig. 7:**
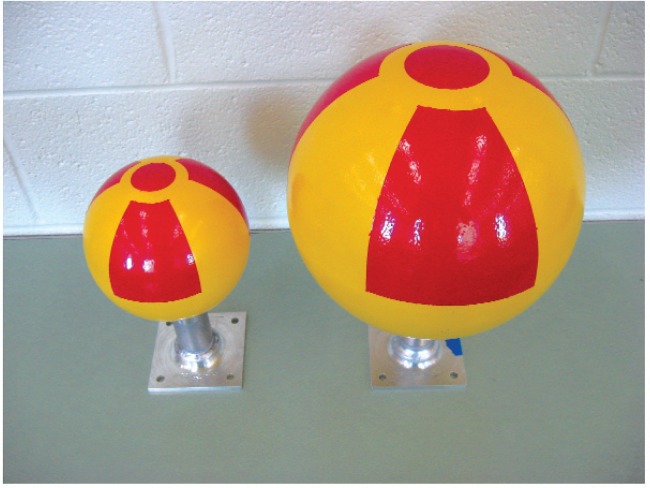
15 cm (6 inch) and 25 cm (10 inch) diameter aluminum spheres with aluminum stems and flanges.

**Fig. 8:**
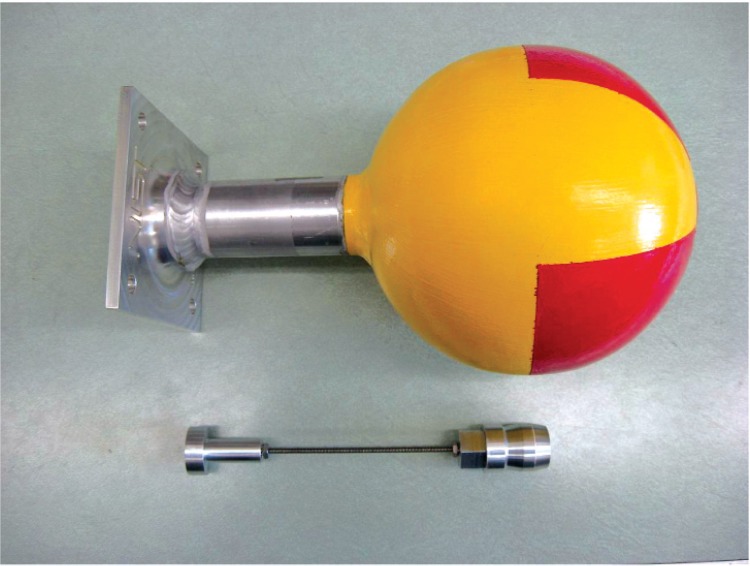
15 cm (6 inch) diameter aluminum sphere with aluminum stem and flange. Next to the sphere is the aluminum holder for the ^57^Co and ^133^Ba sources.

**Fig. 9:**
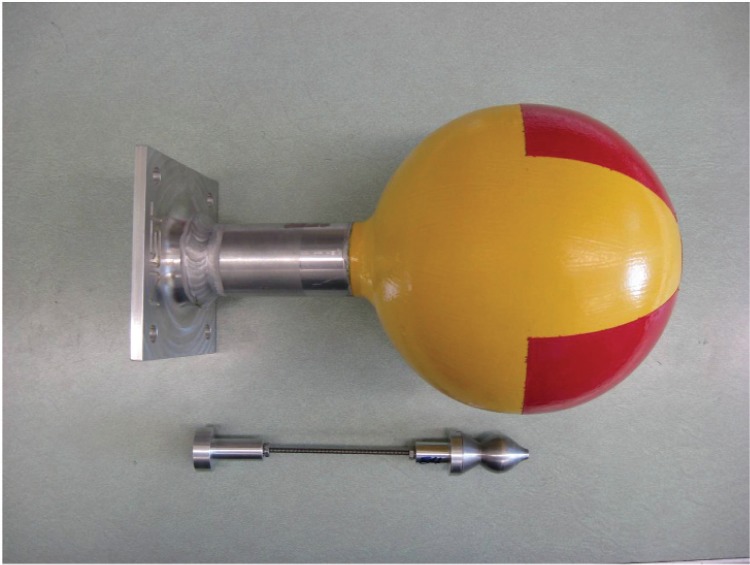
15 cm (6 inch) diameter aluminum sphere with aluminum stem and flange. Next to the sphere is the lead holder for the ^137^Cs source.

**Fig. 10:**
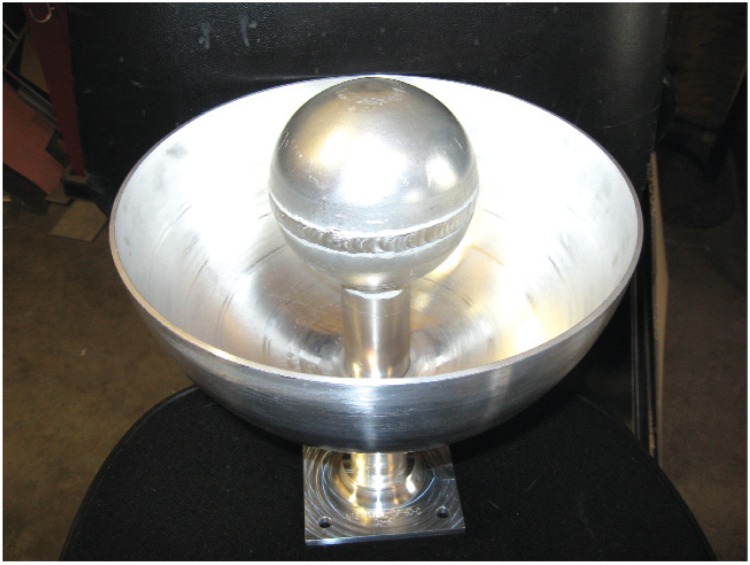
Sphere for holding the ^60^Co pellets inside the 25 cm (10 inch) aluminum sphere.

**Fig. 11:**
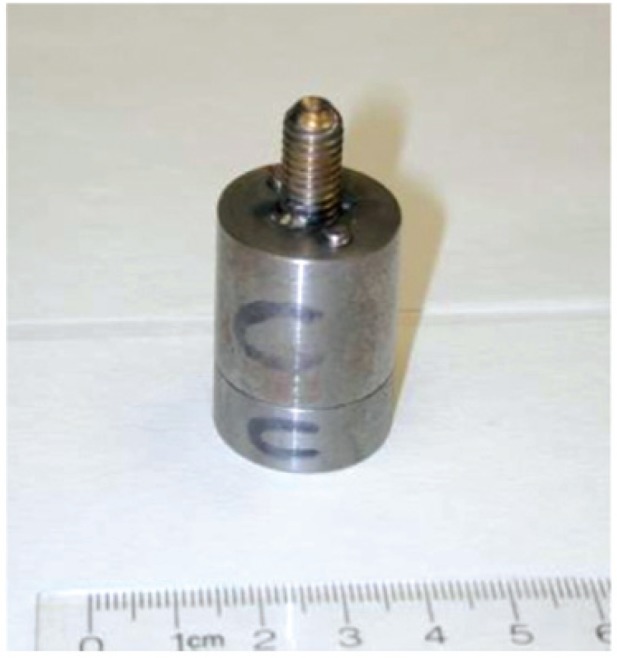
Steel cylinder holding the ^252^Cf seed inside.

Figures 7 to 11 show pictures of the main parts of the different sources. These test sources are shipped in steel pails with locking rings and security seals. The test source in each pail hangs from a slide-in bracket bolted to the underside of the steel cover. The lid and bracket are heavy enough to form a mounting base that may be suitable where vibration or other displacement forces are not a factor.

The 10 cm × 10 cm × 0.6 cm (4 inch × 4 inch × 0.25 inch) flange on the stem has 4 holes for mounting purposes. The spacing of the holes is 7.6 cm (3 inches), center-to-center. Also available are 15 cm × 15 cm (6 inch × 6 inch) slide-in mounting brackets (similar to the slide-in brackets in the pail) that can be bolted to other fixtures.

## 5. Source Characterization

**Fig. 12:**
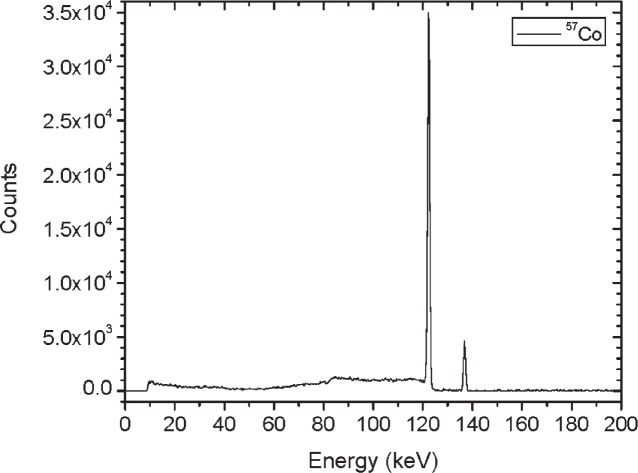
Spectrum for ^57^Co source (Set A) a 90°.

**Fig. 13:**
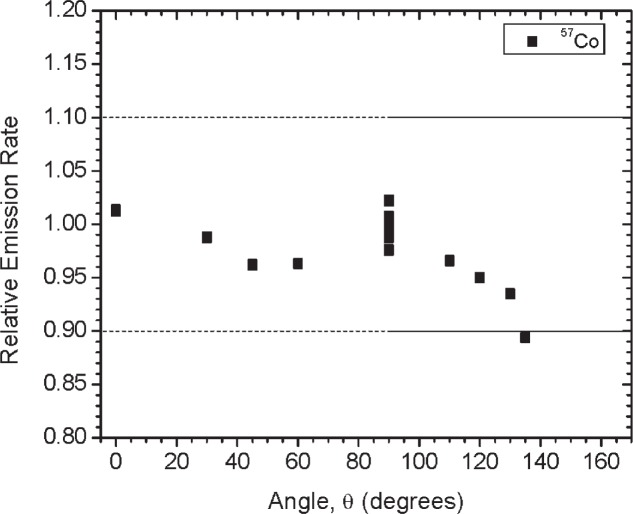
Relative Emission rate of the 122 keV gamma ray line of the ^57^Co source as a function of the angle θ for Set A. The emission rates are plotted relative to the average emission rate measured at θ = 90°. Uncertainties displayed have a coverage factor of *k* = 1.

**Fig. 14:**
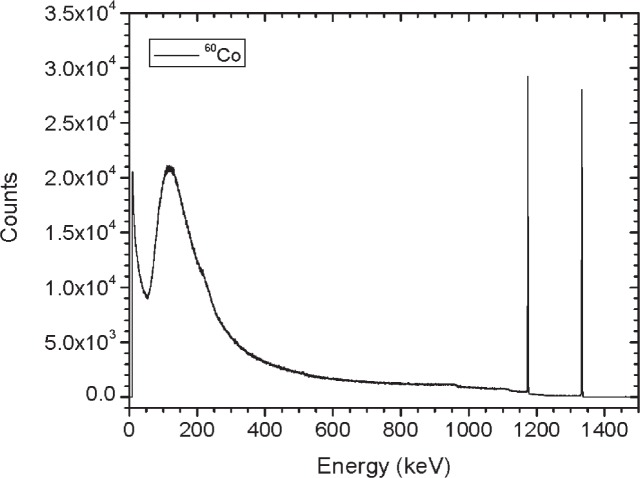
Spectrum for ^60^Co source (Set A) at 90°.

**Fig. 15:**
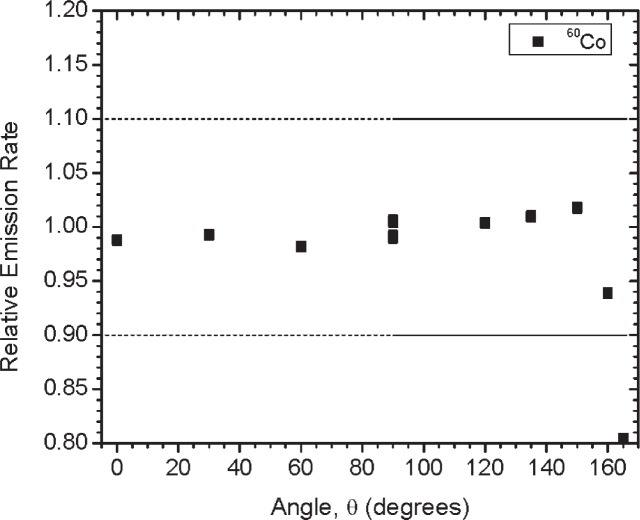
Relative Emission rate of the 1332 keV gamma ray line of the ^60^Co source as a function of the angle θ for Set A. The emission rates are plotted relative to the average emission rate measured at θ = 90°. Uncertainties displayed have a coverage factor of *k* = 1.

**Fig. 16:**
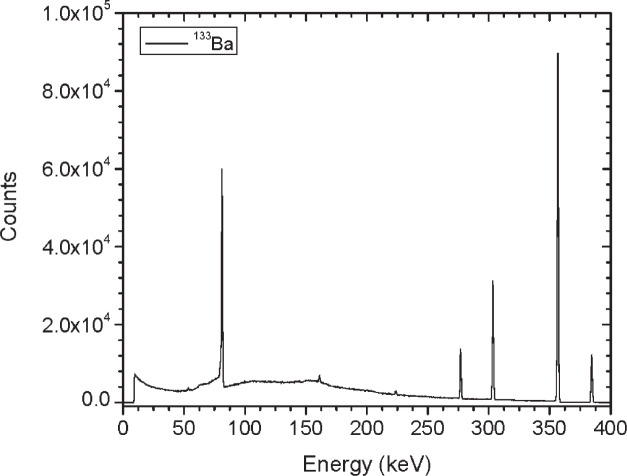
Spectrum for ^133^Ba source (Set A) at 90°.

**Fig. 17:**
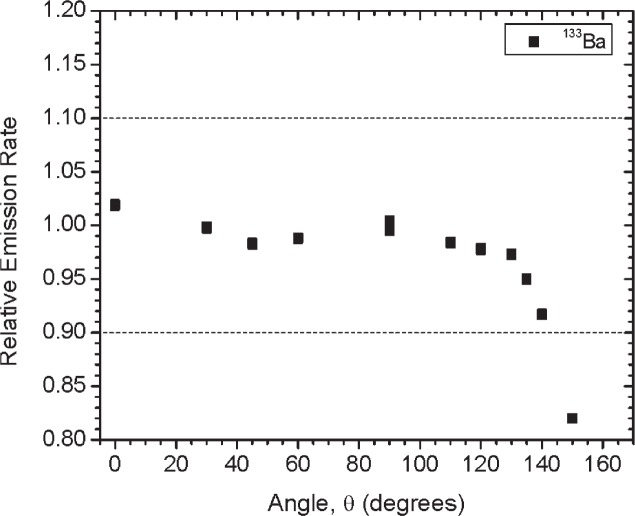
Relative Emission rate of the 356 keV gamma ray line of the ^133^Ba source as a function of the angle θ for Set A. The emission rates are plotted relative to the average emission rate measured at θ = 90°. Uncertainties displayed have a coverage factor of *k* = 1.

**Fig. 18:**
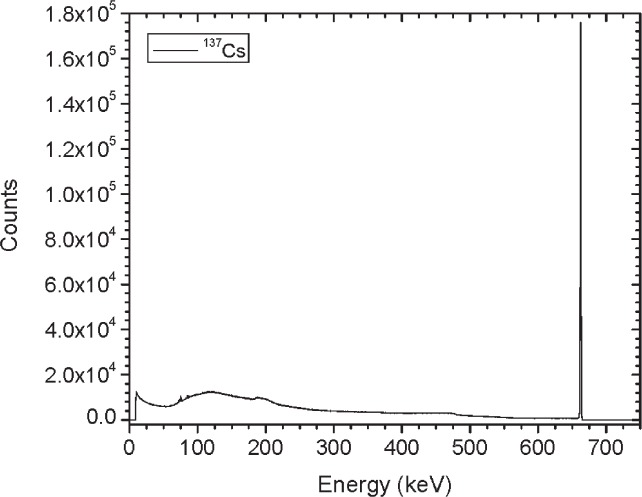
Spectrum for ^137^Cs source (Set A) at 90°.

**Fig. 19:**
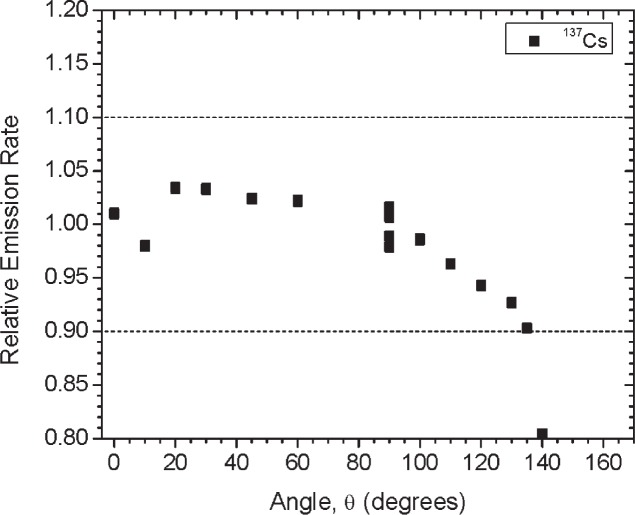
Relative Emission rate of the 662 keV gamma ray line of the ^137^Cs source as a function of the angle θ for Set A. The emission rates are plotted relative to the average emission rate measured at θ = 90°. Uncertainties displayed have a coverage factor of *k* = 1.

Initially, three identical sets of the gamma-ray emitting sources and two sets of the ^252^Cf sources were built and calibrated. Results summarized here are for the first set of sources referred to as Set A. Sources were characterized based on dose rate, gamma-ray emission rate, angular distribution and spectral shape. Table 2 gives a summary of the dose rate and emission rate measurements for each of the sources. For the gamma-ray emitting sources, the gamma-ray emission rates and angular distribution measurements (shown in Figures 12 to 19) were performed using the HPGe L-detector. The main components of uncertainty for the gamma-ray emission rate measurements are the HPGe detection efficiency, the emission rate variability for the y-axis rotation, the source-to-detector distance and the counting statistics.

The gamma-ray emission rate *R*(*E*) is calculated from the acquired energy spectrum using Eq. (1)

$$R(E)=\frac{N(E)}{T\times \varepsilon (E)}\prod_{i}C_{i},$$ (1)

where *N*(*E*) is the number of counts in the full-energy peak, *T* is the measuring time, *ε*(*E*) is the full-energy-peak efficiency and *C_i_* are the correction factors. The decay correction from the calibration source reference time to the middle of the run time is *C_d_*, given by Eq. (2)

$$C_{d}=e^{-\lambda T},$$ (2)

where *λ* is the decay constant *ln*(2)/*T*_1/2_, *T*_1/2_ is the half-life and *T* is the time interval over which the source decays, corresponding to the real (or run) time. The dead time for the gamma-ray measurements was kept below 2 % to avoid pulse pile-up effects.

The uncertainty of the emission rate is obtained using uncertainty propagation and assuming that all measured quantities are independent. The uncertainty for the full-energy-peak efficiency is given by

$$\sigma_{R}=\sqrt{{\left(\frac{\partial R}{\partial N}\right)}^{2}\sigma_{N}^{2}+{\left(\frac{\partial R}{\partial T}\right)}^{2}\sigma_{T}^{2}+{\left(\frac{\partial R}{\partial \varepsilon }\right)}^{2}\sigma_{\varepsilon }^{2}+{\left(\frac{\partial \varepsilon }{\partial \lambda }\right)}^{2}\sigma_{\lambda }^{2}},$$ (3)

where *σ_N_*, *σ_T_*, *σ_ε_*, *σ_λ_* are the uncertainties associated with the quantities *N*(*E*), *T*, *ε*(*E*), *λ*, respectively, assuming that the only correction made is due to source decay. The additional main components of uncertainties, the emission rate variability for the *y*-axis rotation and the source-to-detector distance, were added in quadrature with the other uncertainty components. Efficiency uncertainties range from 0.8 % to up 1.8 %; the uncertainty associated with the emission rate for the *y*-axis of rotation ranges from 0.3 % to up 1.6 % and source-to-detector distance uncertainty range from 0.3 % to up 0.5 %.

For the neutron source, the angular distribution measurements were performed using the integration mode of an Eberline ASP-2e survey meter^2^ equipped with a Helium-3 counter tube embedded in a 20 cm diameter polyethylene sphere. The integration time was one hour or more. The main component of uncertainty for the angular distribution ratio measurements is the counting statistics, the uncertainty for these measurements is 2.5 %. The emission rate calibration procedure and associated uncertainties for the ^252^Cf source are described in Ref. [5].

**Fig. 20:**
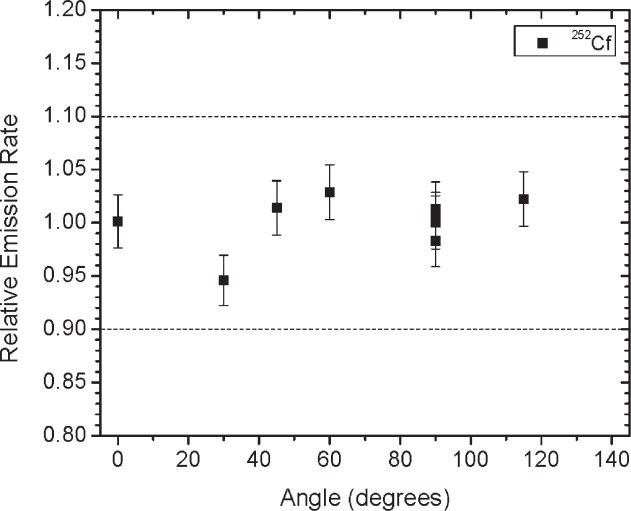
Relative neutron emission rate of the ^252^Cf source as a function of the angle θ. The emission rates are plotted relative to the average emission rate measured at θ = 90° for Set A. Uncertainties displayed have a coverage factor of *k* = 1.

**Fig. 21:**
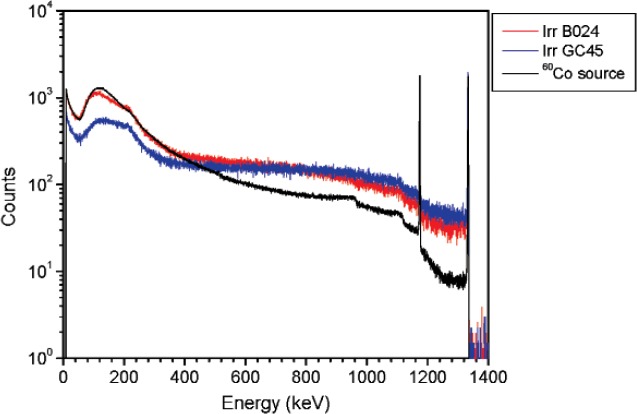
Spectrum for 60Co source at 90° plotted together with spectra for several NIST irradiators. Counts are normalized to 10 000 counts in the 1332 keV photopeak

**Fig. 22:**
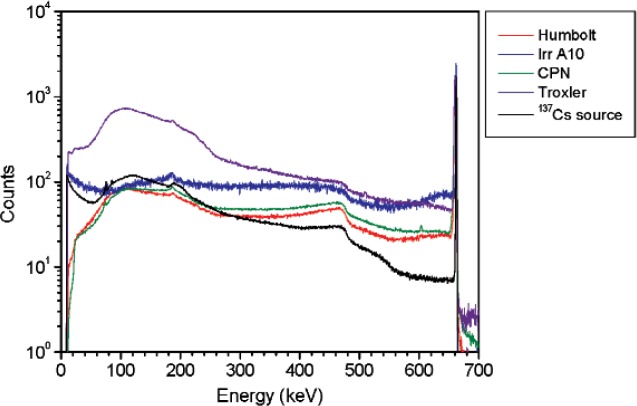
Spectrum for ^137^Cs source at 90° plotted together with spectra for several commercially available density gauges. Counts are normalized to 10 000 counts in the 662 keV photopeak

Figure 1 shows the angles of rotation for the angular distribution measurements. The source spectrum at θ = 90° is shown below for each of the test sources (except ^252^Cf) in Figures 12, 14, 16 and 18. For all spectra, the background was subtracted. The angular distribution of the photon emission rate for the main gamma ray relative to θ = 90° is also shown in Figs. 13, 15, 17, 19 and 20. The uncertainties displayed in these figures have a coverage factor of *k* = 1. From these measurements it can be observed that the gamma-ray emission rates are within ± 10 % from θ = 0° to θ = 135°. The different points at θ = 90° represent the source emission when the source is rotated around the stem. The observed variations of the emission rate with the angle of rotation around the stem can be due to variations in the material thicknesses of the source, changes in source-to-detector distance during measurements and asymmetry of the source mount within the sphere. Variations are larger for the ^137^Cs (1.6 %) and the ^57^Co (1.5 %) sources, this is expected due to the complicated shape of the lead absorber in the ^137^Cs source (see Fig. 9) and the low energy photopeak for ^57^Co. The ^60^Co and the ^137^Cs sources are also plotted together with the irradiators and density gauges, as applicable, in Figs. 21 and 22.

**Fig. 23:**
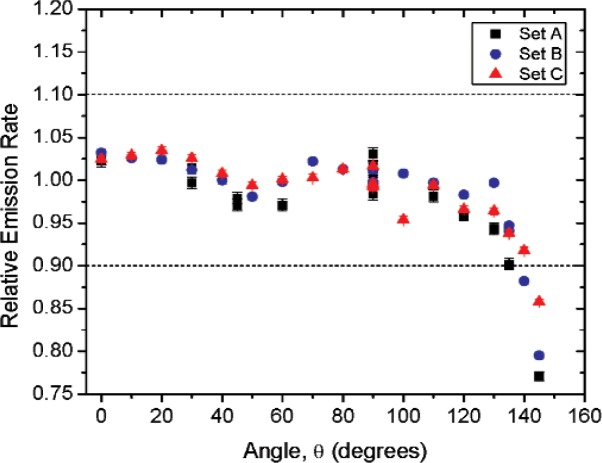
Relative Emission rate of the 122 keV gamma ray line of the^57^Co source as a function of the angle θ for the three sets. The emission rates are plotted relative to the average emission rate measured at θ = 90° for each set. Uncertainties displayed have a coverage factor of *k* = 1.

**Fig. 24:**
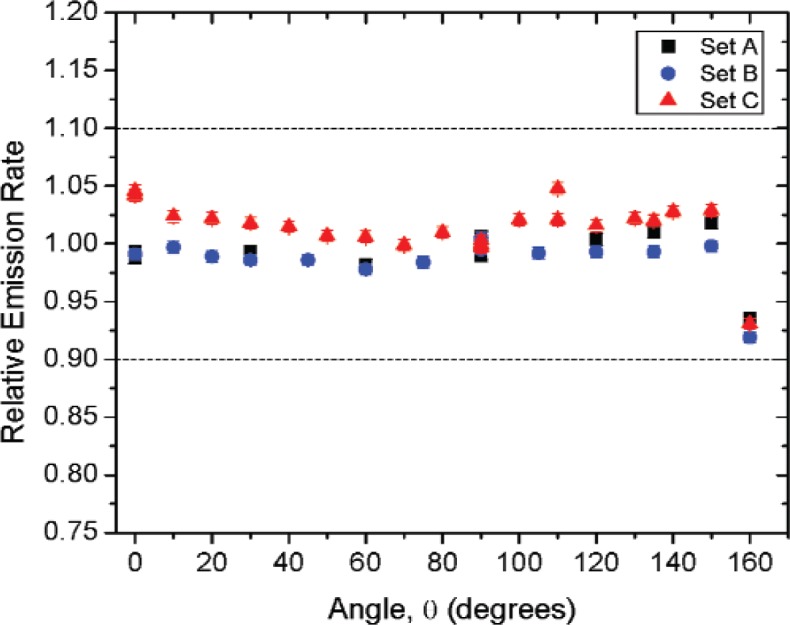
Relative Emission rate of the1332 keV gamma ray line of the ^60^Co source as a function of the angle θ for the three sets. The emission rates are plotted relative to the average emission rate measured at θ = 90° for each set. Uncertainties displayed have a coverage factor of *k* = 1.

**Fig 25:**
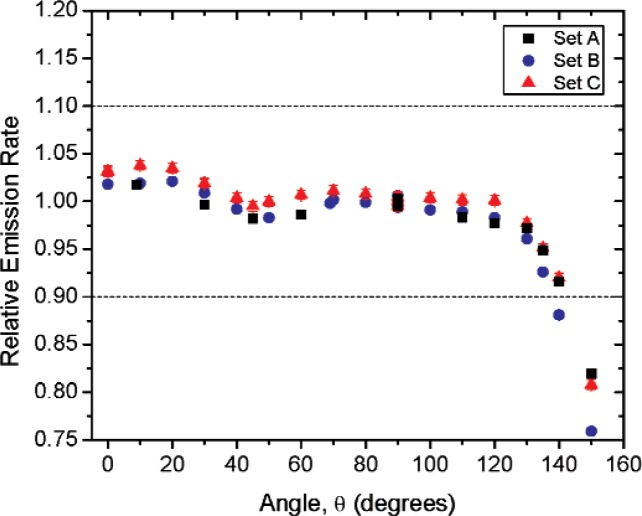
Relative Emission rate of the 356 keV gamma ray line of the ^133^Ba source as a function of the angle θ for the three sets. The emission rates are plotted relative to the average emission rate measured at θ = 90° for each set. Uncertainties displayed have a coverage factor of *k* = 1.

**Fig 26:**
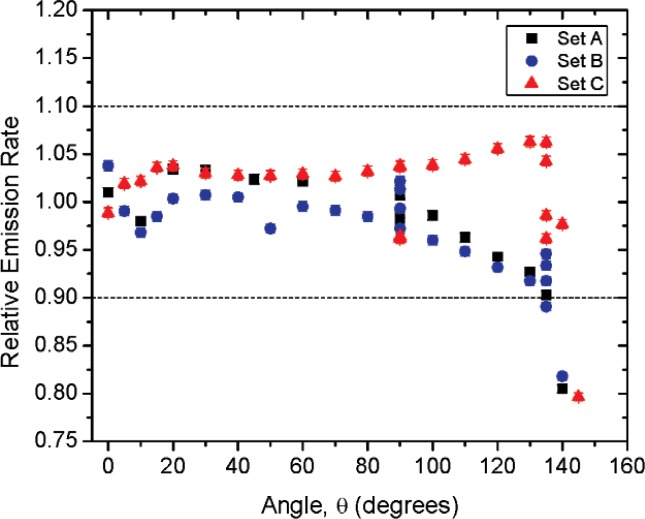
Relative Emission rate of the 662 keV gamma ray line of the ^137^Cs source as a function of the angle θ for the three sets. The emission rates are plotted relative to the average emission rate measured at θ = 90° for each set. Uncertainties displayed have a coverage factor of *k* = 1.

**Fig. 27:**
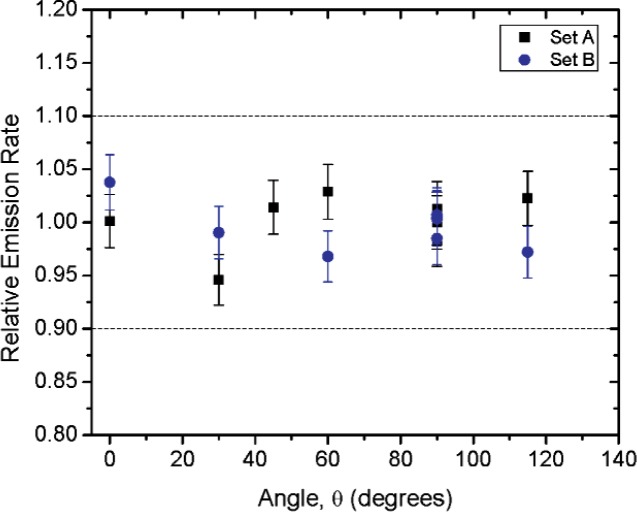
Relative neutron emission rate of the ^252^Cf source as a function of the angle θ for the three sets. The emission rates are plotted relative to the average emission rate measured at θ = 90° for each set. Uncertainties displayed have a coverage factor of *k* = 1.

Variation in the emission rates between sets is less than 5 %. The emission rates variation around θ = 90° for all sets are within the associated uncertainty. For all three sets (two sets for ^252^Cf) the emission rates are within 10 % of the average value at the polar angle θ = 90° for all angles from θ = 0 to θ = 135°, see Figs. 23 to 25 and Fig. 27. The emission rate for the ^137^Cs source as a function of angle from θ = 0 to θ = 135° is the only one that was purposely changed in order to obtained a more uniform angular distribution, see Fig. 26. For this source several measurements were performed by rotating it around the *y*-axis for θ = 135° to check for asymmetries close to the stem position.

## 6. Conclusions

Three sets of radioactive sources (two sets for ^252^Cf) were designed and built at NIST that meet the requirement for testing radiation detection systems in maritime environments. These sources can also be used for testing systems in less demanding environments (i.e., land).

## Figures and Tables

**Table 1:** List of density gauges measured

Manufacturer^2^	Model Number	Activity ^137^Cs (MBq)	Activity AmBe (GBq)	Gamma dose rate at 65 cm (µSv/h)	Neutron dose rate at 65 cm (µSv/h)
CPN	MC-1 DR-P Portable Probe	370	1.9	≈ 6	≈ 2
Troxler	3450 Road Reader Plus	300	1.5	≈ 8	≈ 1
Humboldt Scientific Inc.	5001 EZ	370	1.5	≈ 10	≈ 1

**Table 2:** Summary of the measurement for Set A at 90°

			Gamma Dose Rate (µSv/h) at a distance of:		Neutron Dose Rate (µSv/h) at a distance of:		
Radionuclide	Emission rate* (s^−1^)	Nominal Activity (MBq)+	30 cm	1 m	30 cm	1 m	Description
^57^Co	122 keV Gammas: 1.46 × 10^6^ ± 2.5 %	2.3	≈ 1	≈ 0.2	NA	NA	15 cm (6″) diameter sphere
^60^Co	1332 keV Gammas: 10.18 × 10^6^ ± 2.5 %	85	≈ 50	≈ 7	NA	NA	25 cm (10″) diameter sphere
^133^Ba	356 keV Gammas: 7.15 × 10^6^ ± 2.5 %	14	≈ 7	≈ 1	NA	NA	15 cm (6″) diameter sphere
^137^Cs	662 keV Gammas: 34.2 × 10^6^ ± 2.5 %	185	≈ 40	≈ 6	NA	NA	15 cm (6″) diameter sphere
^252^Cf	Neutrons: 2.2 × 10^4^ ± 2.5 %	0.7	≈ 0.1	ND	≈ 1	≈ 0.2	15 cm (6″) diameter sphere

* Emission rate uncertainty is 2.5 % with a coverage factor of *k* = 1. Reference time for these measurements is 12:00 PM EST June 1, 2009.

+ Nominal activities are listed here because they were part of the design requirements. The emission rates are the only actual measurements reported in this work.

## Footnotes

2 Certain commercial equipment, instruments, or materials are identified in this paper to foster understanding. Such identification does not imply recommendation or endorsement by the National Institute of Standards and Technology, nor does it imply that the materials or equipment identified are necessarily the best available for the purpose.

## References

[1] Lucas L, Pibida L, Unterweger M, Karam L (2005). Gamma-ray Emitting Test Sources for Portal Monitors used for Homeland Security. Radiation Protection Dosimetry.

[2] (2006). ANSI/IEEE N42.43-2006 American National Standard Performance Criteria for Mobile and Transportable Radiation Monitors Used for Homeland Security.

[3] (2006). ANSI/IEEE N42.38-2006 American National Standard Performance Criteria for Spectroscopy-Based Portal Monitors Used for Homeland Security.

[4] (2006). ANSI/IEEE N42.35-2006 American National Standard for Evaluation and Performance of Radiation Detection Portal Monitors for Use in Homeland Security.

[5] Gilliam DM, Yue AT, Dewey MS Calibration of Manganese Bath Relative to ^252^Cf Nu-Bar, private communication.

[6] (2001). ANSI/HPS N13.11-2001 Personnel Dosimetry Performance— Criteria for Testing, Health Physics Society.

